# Paraphenylenediamine (Kala Pathar) Poisoning at the National Poison Control Center in Karachi: A Prospective Study

**DOI:** 10.7759/cureus.8352

**Published:** 2020-05-29

**Authors:** Muhammad Omer Sultan, Muhammad Inam Khan, Rahmat Ali, Umar Farooque, Syed Adeel Hassan, Sundas Karimi, Omer Cheema, Bharat Pillai, Fahham Asghar, Rafay Javed

**Affiliations:** 1 Internal Medicine, Jinnah Postgraduate Medical Center, Karachi, PAK; 2 Internal Medicine, United Medical and Dental College, Karachi, PAK; 3 Neurology, Dow Medical College, Dow University of Health Sciences, Karachi, PAK; 4 Neurology, Dow University of Health Sciences, Karachi, PAK; 5 Internal Medicine, Dow University of Health Sciences, Karachi, PAK; 6 General Surgery, Combined Military Hospital, Karachi, PAK; 7 Neurology, Amrita Institute of Medical Sciences, Kochi, IND; 8 Internal Medicine, Jinnah Hospital, Allama Iqbal Medical College, Lahore, PAK

**Keywords:** paraphenylenediamine, poisoning, cervicofacial edema, human, hair-dye, suicide

## Abstract

Introduction

Suicide by self-poisoning is a common cause of death, especially in the younger population. More specifically, hair-dye poisoning is being increasingly used for suicide. Paraphenylenediamine (PPD), also known as “Kala pathar”, is a highly toxic ingredient present in hair-dye that can cause death. Therefore, this study is designed to assess the demographics, clinical features, laboratory findings, and outcomes of PPD poisoning in patients admitted to the National Poison Control Center in Karachi, Pakistan.

Materials and methods

We conducted a prospective study for a period of six months at the National Poison Control Center, Karachi, Pakistan. A total of eight patients with PPD poisoning with no cardiac, liver, or renal co-morbidities were included in this study. The demographic characteristics, clinical features, laboratory findings, mode of intoxication, and route of intoxication were noted in a proforma. Furthermore, hospitalization time, tracheostomy status, mechanical ventilation status, and mortality rates were also recorded. For continuous variables, the means and SDs were calculated. Whereas for categorical data, percentages were calculated.

Results

In our study, the mean age of the patients was estimated at 25.38 ± 3.77 years. It was deemed that the majority of poisoning cases were intentional in nature (75%). These suicide cases were more commonly observed in young females (75%) who belonged to a low socioeconomic class (87.5%). The preferred route of administration was oral (87.5%). In 87.5% of the patients, the characteristic clinical features such as cervicofacial edema, dysphagia, dysphonia, and stridor were noted. During the later clinical stages of poisoning, clinical features such as rhabdomyolysis (62.5%), chocolate-colored urine (87.5%), hepatitis (75%), and acute renal failure (12.5%) were noteworthy. The mean ± SD of total leukocyte count (TLC), creatine phosphokinase (CPK), aspartate aminotransferase (AST), alanine aminotransferase (ALT), serum creatinine and serum potassium were, respectively, noted at 10,500 ± 3,854.4 cells/mm^3^, 32.87 ± 11.36 IU/L, 1,239.1 ± 1,106.2 IU/L, 776.8 ± 1,149.8 IU/L, 2.125 ± 2.275 mg/dL, and 4.9 ± 1.094 mmol/L. In our patients, the mean intensive care unit stay was 8.25 ± 3.99 days. Emergency tracheostomy was performed in 25% of patients. Mechanical ventilation was required for 50% of our patients. Overall, the mortality rate observed in our study stands at 25%.

Conclusion

PPD poisoning is associated with a high rate of morbidity and mortality. Therefore, it is imperative for physicians to be mindful of the clinical characteristics and treatment options in order to optimally manage such cases of poisoning. In addition, the use of hair-dyes composed of highly lethal PPD should also be banned.

## Introduction

In the younger age group, between the ages of 15 and 44 years, suicide is one of the three most leading causes of death. In developing countries, the rate of suicide has increased very rapidly over the last five decades [1]. Hair-dye poisoning is emerging as a common method of suicide, especially in the low socioeconomic populations of Asia and Africa [2-5]. Hair-dyes contain paraphenylenediamine (PPD), a highly toxic substance, which is an important constituent to produce a variety of shades ranging from golden-blond to black, depending on its concentration [6]. Different hair dyes have varying concentrations of PPD ranging from 0.2%-3.75%. Therefore, hair-dye poisoning is associated with a high fatality rate [2-5].

Pure PPD is in the form of white crystals, which turns brown on exposure to air. It is produced in Germany, Japan, and the United Kingdom [7]. Oral ingestion of PPD can lead to the development of angioneurotic edema, or cardiotoxicity leading to fatal arrhythmias and death within the first six to 24 hours [8]. Smaller doses are usually not fatal and result in clinically relevant angioneurotic edema and hepatitis. Moderate doses can cause acute renal failure within the first week [9]. The first systemic PPD toxicity was diagnosed in the owner of a hair salon by Nott in 1924 [10]. PPD can also cause skin irritation and allergies. Furthermore, its role as a carcinogen in animals has also been described [11].

The aim of this study was to demonstrate the demographics, clinical features, laboratory findings, and outcomes of PPD poisoning patients admitted to the National Poison Control Center in Karachi, Pakistan.

## Materials and methods

Study design

This prospective study took place at the National Poison Control Center Karachi, from 02/01/2013 to 08/01/2013 (for six months). The patient inclusion criterion included the diagnosis of PPD poisoning based on the patient history given by the attendants and clinical examination findings. Patients with known cardiac, liver, and renal co-morbidities were excluded from the study.

Data collection

A total of eight patients met the inclusion/exclusion criteria and were included in this study. The demographic features (age, sex, marital status, socioeconomic status), clinical features (especially cervicofacial edema and color of urine), laboratory findings (complete blood count (CBC), liver function test (LFT), creatine phosphokinase (CPK), glucose, urea, serum creatinine, electrolytes, electrocardiography (ECG), and arterial blood gases), mode of intoxication (accidental or suicidal), and route of intoxication (gastrointestinal system or skin) were noted in a preformed proforma. Hospitalization time, tracheostomy, tracheal intubation, mechanical ventilation, and mortality rates were also noted.

Data analysis

Data were entered and analyzed on Statistical Package for the Social Sciences, version 19 (SPSS Statistics, Chicago, IL). Continuous data were presented as mean and SD values. Whereas categorical data were presented in numbers and percentages.

## Results

The mean age of patients in our study was estimated at 25.38 ± 3.77 years. The majority of the patients (62.5%) were between the ages of 21 and 30 years. A total of six (75%) female and two (25%) male patients were identified. In six patients (75%), the reason for ingestion was mainly attempted suicide. The remaining two (25%) were declared to be accidental. All patients enrolled in our study lived in rural areas where access to healthcare facilities was not available. The poison was taken orally in seven (87.5%) cases and by transdermal route in one (12.5%) case. The demographic features are summarized in Table 1.

**Table 1:** Demographic characteristics of the patients

Parameters	Number of cases	Percentage
Age (years) mean ± SD		
25.38 ± 3.77		
Age (years)		
12–20	02	25%
21–30	05	62.5%
31–40	01	12.5%
Gender		
Male	02	25%
Female	06	75%
Marital status		
Single	03	37.5%
Married	05	62.5%
Socioeconomic status		
High	00	00%
Middle	01	12.5%
Low	07	87.5%
Mode of intoxication		
Suicidal	06	75%
Accidental	02	25%
Route of intoxication		
Oral	07	87.5%
Transdermal	01	12.5%

The clinical features of hair-dye poisoning (pain in the throat, oral erythema, cervicofacial edema, dysphagia, and dysphonia) were present in a majority (87.5%) of patients. Evidence of rhabdomyolysis (muscle aches/tenderness, cola-colored urine, raised CPK levels, myoglobinuria) was present in five (62.5%) cases. Hemodynamic shock and sinus bradycardia were detectable in two (25%) cases. Sinus tachycardia was noted in six (75%) patients. Oliguria/anuria was reported in two (25%) patients, and acute renal failure was inferred in one (12.5%) of the cases. Hepatitis was observed in six (75%) patients. The clinical features of PPD poisoning are summarized in Table 2.

**Table 2:** Clinical features of PPD poisoning PPD, paraphenylenediamine

Clinical features	Number of cases	Percentage
Pain in throat	07	87.5%
Oral erythema	07	87.5%
Cervicofacial edema	07	87.5%
Dysphagia	07	87.5%
Dysphonia	07	87.5%
Difficulty in opening of mouth	07	87.5%
Muscle aches/rigidity	06	75%
Dark urine	07	87.5%
Rhabdomyolysis	05	62.5%
Oliguria/anuria	02	25%
Acute renal failure	01	12.5%
Hyperkalemia	05	62.5%
Hepatitis	06	75%
Hemodynamic shock	02	25%
Sinus bradycardia	02	25%
Sinus tachycardia	06	75%

Regarding the laboratory investigations, the mean ± SD total leukocyte count (TLC), CPK, aspartate aminotransferase (AST), alanine aminotransferase (ALT), serum creatinine, and serum potassium were, respectively, noted at 10,500 ± 3,854.4 cells/mm^3^, 32.87 ± 11.36 IU/L, 1,239.1 ± 1,106.2 IU/L, 776.8 ± 1,149.8 IU/L, 2.125 ± 2.275 mg/dL, and 4.9 ± 1.094 mmol/L (Table 3).

**Table 3:** Laboratory parameters mm^3^, cubic millimeter; IU/L, international units per litre; mg/dL, milligrams per decilitre; mmol/L, millimoles per litre

Laboratory parameters	Mean ± SD	Mode/range
Total leukocyte count (cells/mm^3^)	10,500 ± 3,854.4	5,000/5,000–15,000
Creatine phosphokinase (IU/L)	32.87 ± 11.36	23/23–52
Aspartate aminotransferase (IU/L)	1,239.1 ± 1,106.2	1,400/122–3,250
Alanine aminotransferase (IU/L)	776.8 ± 1,149.8	90/34–3,450
Serum creatinine (mg/dL)	2.125 ± 2.275	0.9/0.5–07
Serum potassium (mmol/L)	4.9 ± 1.094	3.6/3.6–6

The mean intensive care unit stay was 8.25 ± 3.99 days. A majority of patients received gastric lavage, parenteral steroids, sodium bicarbonate, dextrose, and saline. Forced diuresis with furosemide was also used to enhance the excretion of toxins via urine. Tracheostomy was performed in two (25%) patients, and tracheal intubation was required in two (25%) patients. All of the above four (50%) patients were maintained on controlled mechanical ventilation with O_2_ saturation above 95%. Weaning from mechanical ventilation was carried out with the help of pressure-support weaning and T-tube trials. One (12.5%) patient developed acute renal failure 72 hours after the poisoning. Furthermore, a total of two patients (25%) expired. The outcomes of PPD poisoning are summarized in Table 4.

**Table 4:** Outcomes of PPD poisoning PPD, paraphenylenediamine

Outcomes	Number of cases	Percentage
Intensive care unit stay (days) mean ± SD		
8.25 ± 3.99		
Tracheostomy	02	25%
Mechanical ventilation	04	50%
Mortality	02	25%

## Discussion

Hair-dyes that contain PPD are very cheap and easily available everywhere. They are salty, but not bitter in taste which makes it easy to be used as a poison. Elements that compose these hair-dyes include 4% PPD, resorcinol, propylene glycol, ethylenediaminetetraacetic acid (EDTA), sodium, liquid paraffin, cetostearyl alcohol, sodium lauryl sulfate, herbal extracts, preservatives, and perfumes [12].

This study showed that PPD poisoning is more common among females (75%). More specifically, young females between the ages of 21 and 30 years (62.5%) were seen. Furthermore, six (75%) cases were of suicidal intent. All of our patients lived in rural areas and were of low socioeconomic status. The aforementioned findings are similar to the results of various previously conducted studies. In a study conducted by Filali et al., most cases of PPD poisoning were seen in females (77%) between the ages of 15 and 35 years. Furthermore, they also reported a majority (78.1%) of the poisonings being suicidal in nature [13]. Chrispal et al. also identified cases of poisoning involving a similar age-group with female predominance [14]. In our opinion, social and personal conflicts may be the reason for cases of poisoning presenting between the ages of 21 and 30 years.

The typical clinical features encountered in the present study include cervicofacial edema (87.5%), dysphagia (87.5%), chocolate-brown-colored urine (87.5%), pain and/or rigidity of limbs (75%), decreased urine output (37.5%), and convulsions (25%). Kallel et al. reported cases that were dominated by cervicofacial edema (79%), chocolate-brown-colored urine (74%), upper-airway-tract edema (68.4%), and oliguria (36.8%) [15]. Therefore, our findings are quite similar to those of Kallel et al. As observed by Chrispal et al. (69.2%), in our study, cervicofacial edema was the first symptom to develop [14]. The exact cause for this remains unclear. Coma/unconsciousness is another important feature observed in two (25%) of our patients. This was also observed in 26.3% and 20% of patients in studies conducted by Kallel et al. and Akbar et al. [5,15]. Hyperkalemia was observed in five (62.5%) patients. This is comparable to a local study where 26.3% of patients were also reported to have hyperkalemia [16]. Rhabdomyolysis and acute renal failure may be the cause of hyperkalemia. Rhabdomyolysis was observed in 62.5% of patients in our study. Whereas, Kallel et al. noted rhabdomyolysis in all of the patients in their study. In the present study, acute renal failure occurred in 12.5% of patients. This is relatively less compared to 47.4% with Kallel et al., and 40% for Akbar et al. [5,15].

Markers of hepatitis (AST/ALT) were significantly higher in 75% of our patients when compared to the findings of a local study (40%) [16]. It was observed that levels of AST were higher when compared to ALT. It appears that muscle injury due to poisoning leads to a rise in AST.

The management of PPD poisoning is mainly supportive and no specific antidote is available. Dialysis is required in patients who develop acute renal failure, but none of our patients needed dialysis. Respiratory failure is the main threat to life. Therefore, endotracheal intubation, tracheostomy, and assisted ventilation were crucial and lifesaving measures. A study conducted at Nishtar Hospital (Multan) observed a 60% rate of tracheostomy [5]. Suliman et al. observed a tracheostomy rate of 15.8% in their patients [16]. Similarly, tracheostomy was required in 25% of our patients. We observed a mortality rate of 25%. This is similar to the mortality rate of 21.1% for Filali et al. and 20% at Nishtar Hospital in Multan [5,13]. This may depend on the quantity of poison consumed, late arrival of the patient to the hospital, and collaboration of different specialties required for ideal management. Optimal management requires interdisciplinary co-ordination between the disciplines of otolaryngology, anesthesia, nephrology, medicine, and laboratory medicine.

## Conclusions

PPD poisoning is an emerging cause of self-poisoning because of its wide availability at a lower cost. Some countries consider PPD to be a great hazard and have banned its use in hair-dyes. PPD was banned by Germany in the early 1900s, followed by France and Sweden. However, it is still a common component in hair-dyes in Japan, Pakistan, and India. Therefore, our findings should alert the Asian countries. In view of its high rate of mortality, it is important that the medical community should be aware of this poison. Advertisement and sale of this toxic PPD containing hair-dyes should be banned by these governments.
